# Harmonic Scalpel Compared to Conventional Hemostasis in Thyroid Surgery: A Meta-Analysis of Randomized Clinical Trials

**DOI:** 10.1155/2010/396079

**Published:** 2010-02-16

**Authors:** Adrienne L. Melck, Sam M. Wiseman

**Affiliations:** Department of Surgery, Saint Paul's Hospital, University of British Columbia, C303-1081 Burrard Street, Vancouver, BC, Canada V6Z 1Y6

## Abstract

*Background*. The study's aim was to determine whether conventional hemostasis (CH) or the Harmonic Scalpel (HS) results in shorter operative times for thyroidectomy and to evaluate the incidence of postoperative complications with each approach. *Methods*. A literature search was conducted from study inception to September 30, 2008. Included studies randomized thyroidectomy patients to either CH or HS and reported the incidence of postoperative transient recurrent laryngeal nerve dysfunction (RLND) and hypocalcemia. *Results*. Nine RCTs were included. Use of the HS reduced operative time by 23.1 minutes (95% CI = 13.8, 32.33). There was no difference in the incidence of transient RLND (RR = 1.25, 95% CI = .56, 2.76), but a lower rate of transient hypocalcemia with the use of the HS (RR = .69, 95% CI = .51, .92). *Conclusions*. The use of HS in thyroidectomy significantly reduces operative time and is associated with a reduction in postoperative hypocalcemia compared to CH.

## 1. Introduction

There have been few developments in the technical aspects of thyroid surgery since the surgical approach described by Kocher greater than a century ago [1]. Given the significant vascularity of the thyroid gland [2] and the relatively small operative field, meticulous hemostasis has and will always be an important prerequisite for a successful outcome in thyroid surgery. The mainstay for achieving hemostasis in thyroid surgery has been tying and/or clipping of blood vessels, both effective but time-consuming techniques. In the current climate of healthcare constraints and long surgical waiting lists, any methodology that can reduce operative times while maintaining acceptable complication rates warrants investigation. 

The Harmonic Scalpel (Ethicon Endosurgery, Cincinnati, Ohio) was introduced into the surgeon's armamentarium almost two decades ago. Using mechanical vibrations at 55.5 kHz, this device is able to cut and coagulate tissue simultaneously. The proposed advantages of using this device over traditional electrocautery include less lateral thermal tissue injury, a lack of neuromuscular stimulation, and the avoidance of electrical energy transmission either to or through the patient [3]. Since the adoption of the Harmonic Scalpel (HS) into modern surgical practice, its utility for a wide variety of operations has been well documented. For example, a randomized prospective clinical trial demonstrated its ability to diminish blood loss as well as operative time for laparoscopic Nissen fundoplication [4]. 

Over the last decade, many reports have evaluated the utility of the HS for thyroid surgery and the majority of these studies have been carried out at European centers. The investigators have shown similar results regarding reduced operative times with its utilization, but conflicting results regarding other postoperative outcomes such as transient postoperative hypocalcemia and recurrent laryngeal nerve dysfunction (RLND). These complications are relatively uncommon and the number of cases reported in individual studies is limited. Consolidating the data may allow for elucidation of significant associations between HS utilization and postoperative complications. To date, no meta-analysis evaluating the utilization of HS in thyroid surgery has been reported. The purpose of this study was to determine whether conventional hemostasis (CH) or the HS results in shorter operative times for thyroidectomy and to evaluate the incidence of postoperative complications with each approach.

## 2. Methods

### 2.1. Identification of Trials

We sought to identify prospective, randomized clinical trials comparing HS to CH methods (i.e., ties, clips, and/or electrocautery) for thyroidectomy utilizing a computerized literature search. We searched the Cochrane Central Register of Controlled Trials, MEDLINE and EMBASE (January 1, 1995 to September 30, 2008), using the following index terms: thyroidectomy, thyroid surgery, harmonic scalpel, harmonic shears, ultrasonic shears, ultrasonic scalpel, ultrasonic coagulator, ultrasonic dissector, ultrasonic dissection, ultrasonically activated scalpel, ultrasonic scissors, and coagulating shears. In addition, we reviewed the reference lists of retrieved articles, contacted experts in the field, and contacted the major manufacturer of the HS (Ethicon Endosurgery) to determine if they were funding or aware of any trials being conducted using their product. We also searched the proceedings of major endocrine surgery conferences for any reported trials that may not have been published. All studies were considered relevant irrespective of publication status or the language of publication.

### 2.2. Study Eligibility

We restricted our study to adults older than 18 years of age. Only studies comparing traditional open thyroidectomy utilizing CH techniques to thyroidectomy using the HS were considered. Any studies evaluating video-assisted or endoscopic thyroidectomy were excluded. Studies where additional procedures were carried out at the time of thyroidectomy (e.g., lateral neck lymph node dissection) were also excluded, unless these additional procedures were accounted for by subtracting the time for the added procedure from the overall operative time. Thyroid surgery for either benign or malignant histology was included. 

The principal outcome evaluated was the mean operative time, measured in minutes, for total or subtotal thyroidectomies carried out utilizing the two surgical techniques. Although studies could include a combination of total and subtotal thyroidectomies and thyroid lobectomies, they were excluded if they did not report a mean operative time specifically for the total and subtotal thyroidectomies. The secondary outcomes we evaluated were the incidence of transient postoperative RLND and hypocalcemia. Transient RLND was not well defined in most studies. One study defined RLND as transient if vocal cord function recovered within twelve months of the operation [5]. Some studies did not provide an explanation for how transient RLND was diagnosed [6–8]. In the majority of the papers, postoperative laryngoscopy was performed on every patient to assess vocal cord function [5, 9–13]. Transient postoperative hypocalcemia was defined either by biochemical parameters or by clinical symptoms or both. Some studies did not describe how postoperative hypocalcemia was defined. Although studies could report on a variety of secondary postoperative outcomes (e.g., amount and/or duration of wound drainage, postoperative hematoma formation, pain, analgesic requirements, time to hospital discharge, cost-effectiveness), they were excluded if they did not report these two specific outcomes. 

To be included, studies had to be prospective, randomized clinical trials and observational studies were not included in the analysis. Clearly blinding is not feasible in studies evaluating two different surgical techniques, though it was noted if assessors of the postoperative outcomes were blinded to the intervention. 

Regarding data collection and analysis, the two authors (AM and SMW) independently assessed the titles and abstracts of studies retrieved from the literature search and obtained full articles for all those that appeared to satisfy inclusion criteria, ultimately including those that met inclusion criteria after in depth review. The data from those studies were extracted independently by the authors, and any differences were resolved by discussion. The following information was abstracted for each study: year of publication, language of publication, country of origin, study design (including details on randomization, blinding, allocation concealment, intention-to-treat analysis, and losses to follow-up), provision of industrial support for the study, reason for ineligibility if the study was ultimately excluded, number of patients enrolled in each study arm, indication for thyroidectomy, type of thyroidectomy carried out (e.g., partial versus total versus subtotal), details regarding type of HS and CH utilized (ties versus clips versus electrocautery), mean operative time for total and subtotal thyroidectomies in each group, number of cases of transient and permanent postoperative hypocalcemia (either symptomatic or biochemical), number of cases of postoperative transient or permanent RLND, and number of cases of postoperative hematoma formation. Study validity is presented qualitatively though no formal validity score was assigned.

### 2.3. Statistical Analysis

For the primary outcome, the meta-analysis evaluated the weighted mean difference in operative times between thyroidectomy groups (HS versus CH) and the standard deviation of the difference from individual studies using the METAN command in STATA 9.2 (StataCorp, College Station, Texas). In one study, the data regarding operative times was not reported as a mean with standard deviation, but after correspondence with the authors, the data was provided in such a format as to allow inclusion in the analysis [11]. In 2 cases, attempts to contact the authors were unsuccessful and thus these papers could not be included in the analysis, though they had otherwise met inclusion criteria [14, 15]. Significant heterogeneity across studies was noted; thus a pooled estimate of the difference in operative time was generated using a random effects model [16]. A sensitivity analysis excluding the two studies that disclosed financial support from the HS manufacturers was also carried out.

For the secondary outcomes of postoperative RLND and hypocalcemia, results are presented as risk ratios (RRs). The fixed effects model was utilized to obtain the summary estimates of the logRR from the group of studies. We did not proceed to a random effects model once the fixed effects analysis did not reveal any significant heterogeneity (Q statistic).

Publication bias was assessed with Begg's and Egger tests and Begg's funnel plot [17, 18]. A *P*-value of <.05 was considered statistically significant.

## 3. Results

**Figure 1:**
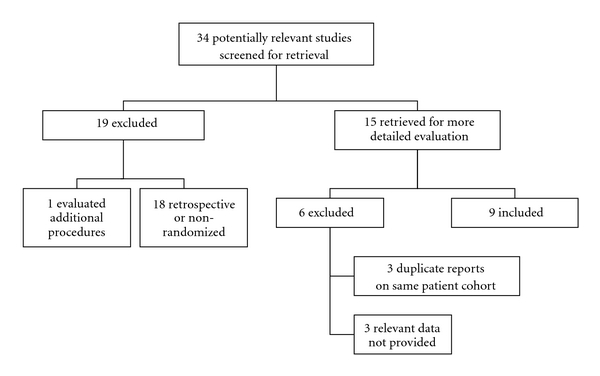
Flow diagram showing the number of studies initially identified and the reasons for study exclusion.

Thirty-four studies that potentially met inclusion criteria were identified from the literature search. After abstract screening, 19 were excluded for variety of reasons. Of the 15 that were reviewed in depth, 6 were excluded, leaving 9 studies that were incorporated into the meta-analysis.  Figure 1 depicts a flow diagram of the study selection process and Table 1 summarizes the characteristics of the studies included in the meta-analysis. There were no incidents of author disagreement in either the study selection or data extraction phase

**Figure 2:**
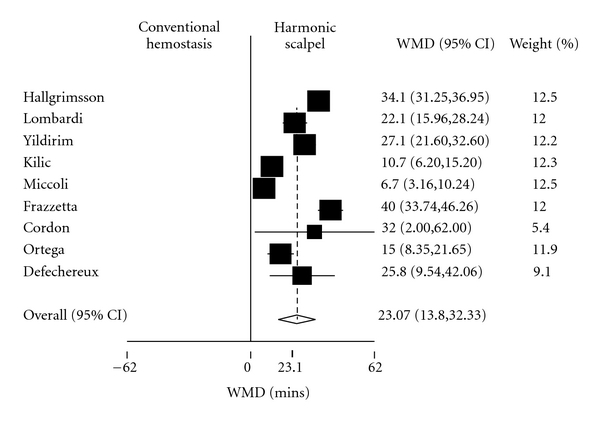
Forest plot depicting individual and pooled weighted mean difference (WMD) in operative times with 95% confidence intervals.

**Figure 3:**
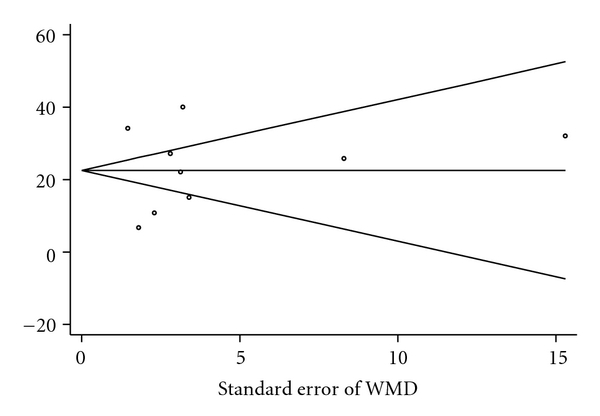
Begg's funnel plot with pseudo 95% confidence limits.

Regarding the primary outcome of mean operative time, the pooled estimate of the weighted mean difference (WMD) in operative time obtained from a random effects model was 23.1 minutes (95% CI = 13.8, 32.33). This was statistically significant, with a *P*-value of <.001 (Figure 2). The *χ* ^2^ test for heterogeneity was significant with a *P*-value of <.001. Tests for publication bias were not statistically significant (*P* = .97). See Figure 3 for Begg's funnel plot.

**Figure 4:**
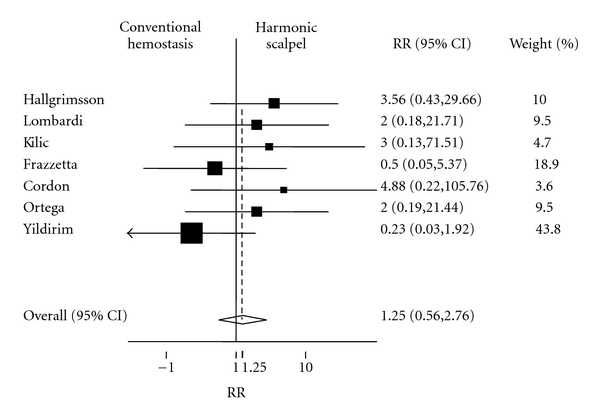
Forest plot depicting individual and pooled risk ratios (RRs) with 95% confidence intervals (CIs) for transient postoperative recurrent laryngeal nerve dysfunction.

Regarding secondary outcomes, the pooled estimate and 95% confidence interval of the relative risk of postoperative transient RLND from a fixed effects model was 1.25 (*P* = .59; 95% CI = 0.56, 2.76). Thus, there is a trend toward an increased risk of transient RLND with the use of HS, but the overall number of cases of this was small and this was not a statistically significant finding (see Figure 4). Two studies reported no cases of transient RLND [10. 12] and were excluded from this analysis. The *χ* ^2^ test for heterogeneity was not significant (*P*-value =  .51); thus we did not proceed to a random effects analysis.

**Figure 5:**
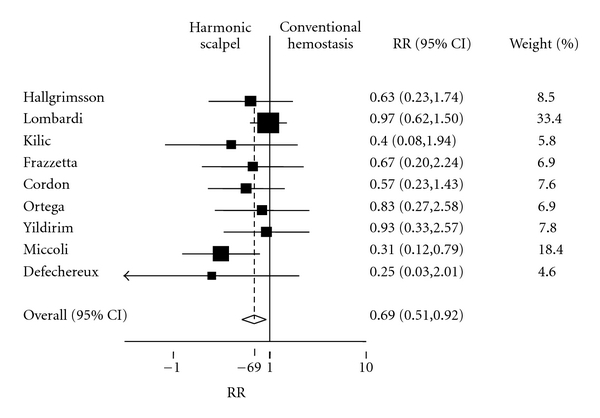
Forest plot depicting individual and pooled risk ratios (RRs) with 95% confidence intervals (CIs) for transient postoperative hypocalcemia.

The pooled estimate and 95% confidence interval of the relative risk of postoperative transient hypocalcemia from a fixed effects model was 0.69 (*P* = .01; 95% CI = 0.51, 0.92). Thus, there was a statistically significant reduced risk of transient postoperative hypocalcemia with the use of HS (see Figure 5). The *χ* ^2^ test for heterogeneity was not significant (*P*-value =  .53); so a random effects analysis was not carried out.

A sensitivity analysis excluding studies with industry support revealed an even greater reduction in operative time with use of the HS (25 minutes; 95% CI = 16.3, 33.62). Interestingly, there were a total of 3 cases of postoperative hematoma in the CH group and 1 in the HS group (Table 1) suggesting a trend toward a lower incidence of this serious postoperative complication with the HS. However, the overall numbers are too small to draw any meaningful conclusions.

The quality of the studies was assessed based on the following criteria: appropriateness of randomization, allocation concealment, blinding of patients, blinding of outcome assessors, utilization of intention-to-treat analysis, and a description of any patients that were lost to follow-up. In most cases, these parameters were not specified and thus the methodological quality of the included studies could only be deemed as fair. These results are presented in Table 2.

## 4. Discussion

Utilization of the HS for total and subtotal thyroidectomy significantly reduced operative time compared to CH techniques by greater than 23 minutes (*P*-value <.001). Furthermore, there was a 31% decreased risk of transient postoperative hypocalcemia with HS utilization compared to CH techniques (pooled RR = 0.69, *P*-value =  .01) and there was also no statistically significant difference in the risk of transient postoperative RLND between the two groups (pooled RR = 1.25, *P*-value =  .59). We conclude that not only is HS utilization for total thyroidectomy significantly faster than the conventional approach, with acceptable postoperative complication rates, but also it may even protect against the development of transient postoperative hypocalcemia.

All of the studies uniformly report decreased operating time with the use of an HS. This is not a surprising observation, given that the same outcome has been reported repeatedly for a variety of other surgical procedures [19–21]. With the exception of a single Mexican study, all of the reports were from European centers. There is no reason to believe that the patients requiring thyroid surgery are any different in Europe than in North America and thus we believe that our results are generalizable to other patient populations. From the literature search, two reports from U.S. centers evaluating HS use for thyroidectomy were identified but excluded because of their retrospective study design. Both of these studies also found the HS to be safe and time-saving [22, 23]. 

All studies reported an increased risk of postoperative hypocalcemia with conventional hemostasis techniques, though only one report had a large enough cohort for the association to be statistically significant [12]. Though the mechanism is not fully understood, transient hypocalcemia observed after total thyroidectomy is believed to be related to traumatization of the parathyroid glands, which are anatomically intimately related to the thyroid gland and share its blood supply. We speculate that use of the HS may facilitate dissection of the parathyroid glands in a plane farther away from the parathyroid gland capsule, thus reducing the chance of damaging their blood supply, directly or indirectly, with either mechanical forces or electrical currents. Thus, this finding of reduced transient postoperative hypocalcemia with HS utilization does seem biologically plausible and highlights an important rationale for conducting the meta-analysis. When an outcome is relatively uncommon, individual studies may all trend toward that same outcome though none may have the power to support statistical significance, but calculating a pooled estimate may allow for the determination of a statistically significant association. It is difficult to draw any conclusions regarding permanent hypoparathyroidism and HS utilization. Permanent hypoparathyroidism is a rare complication of thyroidectomy, and there were only three reported cases of this among the nine studies, two of which occurred in the CH group and one in the HS group (Table 1). 

The complication of RLND after thyroidectomy is also an extremely uncommon occurrence. Included studies had conflicting results in terms of the risks of RLND with HS utilization compared to CH, and all reported either very few or no cases of this complication. In the current meta-analysis, there were only twenty-two incidents of transient RLND out of 822 total thyroidectomies (.03%) or 1,644 nerves at risk (.01%). Given that HS has been shown to cause less collateral thermal injury than conventional electrocautery, we would expect to see less RLND in the HS group. Unfortunately, the numbers in this analysis are too small to generate any meaningful conclusions. Only one case of permanent RLND occurred in a patient who underwent the CH technique. The time cutoff to differentiate between transient and permanent RLND was not well defined in the studies, but most investigators did use postoperative laryngoscopy in all patients to document vocal cord paralysis. 

 Regarding the internal validity of included studies, one must accept that for studies evaluating surgical techniques, blinding of the surgeon is not possible. However, patients can be blinded to the procedure they have undergone to minimize reporting bias when evaluating postoperative outcomes such as symptomatic hypocalcemia or pain. Furthermore, those individuals evaluating outcomes (operative time, RLND, hypocalcemia) can also be blinded to the intervention to reduce observation bias, and this was only explicitly carried out in a single study [9]. Ideally, authors should also give a detailed description of their randomization procedures, allocation concealment, and use of intention-to-treat analysis, which was not consistently reported in the studies included in this meta-analysis. To assure internal validity, future randomized studies evaluating this question should include details addressing these issues. The quality of a meta-analysis is only as good as the reports from which it is derived, and so our study is inherently limited by the methodological limitations of the included reports.

 No tests of publication bias were statistically significant. Begg's funnel plot for the pooled estimate of the WMD in operative time did exhibit some asymmetry, but this was not statistically significant. The asymmetry was likely a result of between-study heterogeneity (tau-squared = 175.88). When between-study heterogeneity is large and when the number of included studies is small, none of these tests to detect publication bias work well. Though all studies found that thyroidectomy was faster with the HS, they were quite heterogeneous in terms of the baseline length of time required to carry out a conventional thyroidectomy (range from 46.7 to 168.8 minutes). This observed difference in time required to carry out the same operation is quite striking. The heterogeneity may have been due to the size of the gland that was being resected, which was not clearly defined in all studies. In addition, all of the thyoidectomies in the Hallgrimsson study, which reported the longest mean operative time for conventional thyroidectomy, were carried out for Graves' thyrotoxicosis, wherein the vascularization of the thyroid gland can be very extensive. In contrast, the majority of thyroidectomies in the study reporting the fastest mean operative time excluded patients with Graves' disease or extensive goiters [12].

 One must consider whether or not benign versus malignant thyroid pathology affected our results. All of the studies incorporated in the meta-analysis excluded patients requiring either a central or lateral compartment lymph node dissection; thus this could not have played a role in operative time or incidence of postoperative hypocalcemia. Of the 9 studies, 4 excluded malignant disease (7, 10, 11, 13), 3 had no significant difference in the proportion of malignant cases between the HS and CH groups (5, 6, 9), 1 only included low-risk T1N0M0 papillary thyroid cancers (12), and 1 did not clearly outline the pathologies. Given this, we do not feel that thyroid pathology is confounding our results for the primary or secondary outcomes. 

Another consideration when interpreting the results of the current meta-analysis is that surgeons who conduct these trials may have significantly more experience with the HS than the average thyroid surgeon, and the timesaving effect of the HS might be exaggerated compared to what a less-familiar surgeon would experience when first adopting its use into their practice.

Future prospective, randomized trials of larger patient cohorts with more detailed and uniform definitions of postoperative complications, randomization procedures, intention-to-treat analyses, and blinding of outcome assessors are needed to draw more meaningful conclusions with regard to the influence of HS utilization on complications after total or subtotal thyroidectomy. In addition, cost-effectiveness analyses to determine whether the costs saved from the reduced time spent in the operating theater outweigh the added cost of the HS scalpel would also be important. Several of the studies did report reduced overall cost associated with the HS [7, 8, 10] while another reported no difference in overall costs when comparing the two techniques [5]. Other benefits seen with HS demonstrated in these studies included less operative bleeding [6, 8–12], fewer cases of postoperative hematoma formation [7], fewer ties used [6, 8, 9, 13], less drain utilization [13], less postoperative pain or analgesic requirements [10, 12], and smaller incisions [6]. The impact of a recently introduced, smaller handheld HS on thyroid surgery outcomes also warrants further study. Reports of the use of another vessel sealing technology, the Ligasure (Covidien, Boulder, Colorado), for thyroid surgery have emerged in the recent literature, and comparisons between this device and the HS would also be of interest. From the current study, we are able to definitively conclude that not only does the use of the HS significantly decrease operative time compared to CH techniques with ties, clips, and/or electrocautery but it is also safer in terms of reducing the incidence of transient postoperative hypocalcemia.

## Figures and Tables

**Table 1:** Characteristics of included studies.

						Mean OR					
Author	Year	Country	Industry	CH	#	Time (min) for	Transient	Permanent	Transient	Permanent	Postoperative
			funding	techniques	patients	TT/ST (SD)	RLND	RLND	hypocalcemia	hypocalcemia	hematoma
Hallgrimsson [11]	2008	Sweden	No	Electrocautery	CH = 24	CH = 168.8 (4.8)	CH = 1	CH = 0	CH = 7	CH = 1	CH = 0
				ligatures clips	HS = 27	HS = 134.7 (5.6)	HS = 4	HS = 0	HS = 5	HS = 0	HS = 0
Lombardi [5]	2008	Italy	No	Electrocautery	CH = 100	CH = 75.2 (23.5)	CH = 1	CH = 0	CH = 29	CH = 0	CH = 1
				ligatures	HS = 100	HS = 53.1 (20.7)	HS = 2	HS = 0	HS = 28	HS = 0	HS = 1
Yildirim [13]	2008	Turkey	No	Electrocautery	CH = 54	CH = 105 (16)	CH = 5	CH = 1	CH = 7	CH = 1	CH = 0
				ligatures	HS = 50	HS = 77.9 (12.5)	HS = 1	HS = 0	HS = 6	HS = 1	HS = 0
Kilic [6]	2007	Turkey	No	Electrocautery	CH = 40	CH = 57.8 (12)	CH = 0	CH = 0	CH = 5	CH = 0	CH = 0
				ligatures	HS = 40	HS = 47.1 (8.2)	HS = 1	HS = 0	HS = 2	HS = 0	HS = 0
Miccoli [12]	2006	Italy	Yes	Electrocautery	CH = 50	CH = 46.7 (10.8)	CH = 0	CH = 0	CH = 16	CH = 0	CH = 0
				ligatures	HS = 50	HS = 40 (6.8)	HS = 0	HS = 0	HS = 5	HS = 0	HS = 0
Frazzetta [8]	2005	Italy	No	Electrocautery	CH = 60	CH = 96 (17)	CH = 2	CH = 0	CH = 6	CH = 0	CH = 0
				ligatures	HS = 60	HS = 56 (18)	HS = 1	HS = 0	HS = 4	HS = 0	HS = 0
Cordon [9]	2005	Mexico	Yes	Electrocautery	CH = 12	CH = 136 (37)	CH = 0	CH=0	CH=9	CH=0	CH = 0
				ligatures clips	HS = 7	HS = 104 (29)	HS = 1	HS = 0	HS = 3	HS = 0	HS = 0
Ortega [7]	2004	Spain	No	Ligatures	CH = 57	CH = 101 (16)	CH = 1	CH = 0	CH = 6	CH = 0	CH = 2
					HS = 57	HS = 86 (20)	HS = 2	HS = 0	HS = 5	HS = 0	HS = 0
Defechereux [10]	2003	Belgium	No	Electrocautery	CH = 17	CH = 96.5 (28.9)	CH = 0	CH = 0	CH = 4	CH = 0	CH = 0
				Ligatures clips	HS=17	HS = 70.7 (18.3)	HS=0	HS=0	HS=1	HS=0	HS=0

TOTAL					CH = 414		CH = 10	CH = 1	CH = 89	CH = 2	CH = 3
					HS = 408		HS = 12	HS = 0	HS = 59	HS = 1	HS = 1

CH: conventional hemostasis; OR: operative; HS: harmonic scalpel; TT: total thyroidectomy; ST: subtotal thyroidectomy; SD: standard deviation; RLND: recurrent laryngeal nerve dysfunction.

**Table 2:** Study validity.

Author	Randomization		Concealed	Patients	Outcome assessors	Intention- to-treat	Patients LTFU?
	Done Adequate		allocation	blinded	blinded	Analysis	
Hallgrimsson	Yes	UC	UC	UC	UC	Yes	UC
Lombardi	Yes	UC	UC	Yes	UC	UC	UC
Yildirim	Yes	UC	UC	UC	UC	UC	UC
Kilic	Yes	UC	UC	UC	UC	UC	UC
Miccoli	Yes	UC	UC	Yes	UC	UC	UC
Frazzetta	Yes	UC	UC	UC	UC	UC	UC
Cordon	Yes	Yes	UC	UC	Yes	UC	UC
Ortega	Yes	UC	UC	UC	UC	UC	UC
Defechereux	Yes	Yes	UC	UC	UC	UC	UC

UC: unclear; LTFU: loss to follow-up.
